# Restoration of an Asphyxiated Infant

**Published:** 1881-03

**Authors:** 


RESTORATION OF AN ASPHYXIATED INFANT.—In a communication to the Academie des Sciences M. Goyraud calls to mind that M. Gustave Le Bon, in a note published in the *Comptes-Rendus* of 1872, indicated as a certain method of recalling to life young animals that had become asphyxiated the plunging them into a bath the temperature of which was gradually raised from 38° to 48° C. M. Goyraud has recently had occasion to employ this means for a new born infant which had been delivered by the forceps. When the infant was extracted the movements of the heart had entirely ceased, and various means for restoring animation, including artificial respiration, were persevered in for nearly two hours. No sign of life appearing, the infant, already become cold, was plunged into a bath heated from 45° to 50° C. (113° to 114° F) Thirty seconds had hardly elapsed when the first inspiration was observed to take place, and was quickly followed by free respiration, the infant in the course of five minutes having become full of life.—*Gaz. Hebd.*, January; *Medical Times and Gazette.*

